# Resveratrol improves Gly-LDL-induced vascular endothelial cell apoptosis, inflammatory factor secretion and oxidative stress by regulating miR-142-3p and regulating SPRED2-mediated autophagy

**DOI:** 10.18632/aging.202546

**Published:** 2021-02-17

**Authors:** Wenjun Sha, Meizhi Liu, Dusang Sun, Junhui Qiu, Bilin Xu, Lin Chen, Tian Shen, Cheng Chen, Hongping Wang, Cuiping Zhang, Tao Lei

**Affiliations:** 1 Department of Endocrinology, Putuo Hospital, Shanghai University of Traditional Chinese Medicine, Shanghai 200062, China

**Keywords:** miR-142-3p, SPRED2, Gly-LDL, autophagy

## Abstract

Background: Resveratrol improves cell apoptosis and tissue damage induced by high glucose, but the specific mechanism is unknown.

Methods: This is a basic research. We performed cell transfection, real-time fluorescence quantitative PCR (qPCR), flow cytometry, immunofluorescence, western blot, enzyme linked immunosorbent assay (ELISA) and cell viability assay to analyze cell viability, cell cycle, cellular oxidative stress, intracellular inflammatory factors and autophagy activities *in vitro*. Meanwhile, dual luciferase reporter assay was conducted to explore the influence of miR-142-3p and sprouty-related EVH1 domain 2 (SPRED 2) on human glycated low-density lipoprotein (Gly-LDL)-induced vascular endothelial cell apoptosis, inflammatory factor secretion and oxidative stress.

Results: Resveratrol inhibited the expression of miR-142-3p in human umbilical vein endothelial cells (HUVECs) induced by Gly-LDL in a dose-dependent manner, and the overexpression of miR-142-3p reverses the effect of resveratrol on the proliferation, apoptosis, secretion of inflammatory factors, oxidative stress, and autophagy. The dual-luciferase report analysis found a negative regulatory relationship between miR-142-3p and SPRED2. Inhibition of SPRED2 reversed the effects of resveratrol on Gly-LDL-induced HUVECs proliferation, apoptosis, inflammatory factor secretion and oxidative stress, and reversed the effects of resveratrol on Gly-LDL-induced HUVECs autophagy.

Conclusion: miR-142-3p promotes the development of diabetes by inhibiting SPRED2-mediated autophagy, including inducing cell apoptosis, aggravating cellular oxidative stress and secretion of inflammatory factors, and resveratrol improves this effect.

## INTRODUCTION

With the improvement of people’s living standards and the continuous change of lifestyles, diabetes has become one of the most common chronic diseases in the world. As the most common metabolic disease, type 2 diabetes (T2DM) is mainly caused by pancreatic β-cell dysfunction and insulin resistance [1]. Faced with the prevalence of T2D, this popularity is increasing every year and getting younger and younger. Although researchers and doctors have actively prevented and treated T2D, the disease still cannot be effectively treated due to the limitations of various therapeutic drugs and methods [2]. In addition, the vascular endothelial repair ability was damaged by the high-sugar environment and the complications such as cardiovascular disease had caused serious threats to the life and health of patients [3]. However, there are still no drugs and methods that can effectively prevent or treat diabetes complications.

Vascular endothelial cells are a layer of monocytes located between the bloodstream and the tissue of the vessel wall [4]. Many studies have found that vascular endothelial cells secrete a variety of vasoactive substances through multiple secretion methods (including autocrine, endocrine and paracrine pathways) to play the functions of regulation, anti-inflammatory and protection [5]. It is known that the etiology and main pathogenesis of diabetic vascular disease is the damage of vascular endothelial cells caused by high glucose, which is mainly manifested by excessive apoptosis and dysfunction of vascular endothelial cells [6]. The damaged vascular endothelial cells make the blood vessels lose their protective effect, and eventually gradually aggravate the development of diabetes.

Meanwhile, the high-sugar environment reduces the nutritional factors released by vascular endothelial cells, breaking the steady state of vascular balance, and ultimately lead to a series of cardiovascular events [7]. Notably, some studies have shown that resveratrol has good anti-inflammatory, antioxidant, cardiovascular protection and anti-cancer effects [8]. Consistent with these findings, another study recognized that resveratrol can significantly improve cell apoptosis and tissue damage induced by high glucose. However, the molecular mechanism is not understood.

As a type of small non-coding RNA, miR-142-3p plays a key regulatory role in the body's physiological and pathological processes [9]. Studies have shown that down-regulating the expression of miR-142-3p improves nerve tissue damage under conditions of hypoxia and hypoglycemia [10]. Meanwhile, up-regulated miR-142-5p inhibits the PTEN pathway and cell autophagy in diabetic nephropathy models. SPRED2, the downstream target of miR-142-3p, activates cell autophagy and other pathways. Autophagy is a protective mechanism of cells. When foreign bodies or nutrients are deficient in the cell, the autophagy pathway is activated to form autophagosomes with double or multi-membrane vesicles to protect the cells from damage [11]. Many researches have shown that autophagy activated by SPRED in cells plays an important protective role in diabetes and high glucose-induced damage [12]. However, its mechanism of action is unclear.

In summary, in order to further study the pathogenesis of high glucose environment and diabetes's vascular damage, we propose the following hypothesis: miR-142-3p may inhibit SPRED2-mediated autophagy and promote the development of diabetes, including inducing cell apoptosis and cell death, and increasing of cell oxidative stress and secretion of inflammatory factors. Resveratrol can improve blood vessel damage caused by high glucose through regulating autophagy and cell inflammation.

## RESULTS

### Resveratrol inhibits the high expression of miR-142-3p in HUVEC induced by Gly-LDL in a dose-dependent manner, and promotes SPRED2 expression and cell proliferation

**Figure 1:**
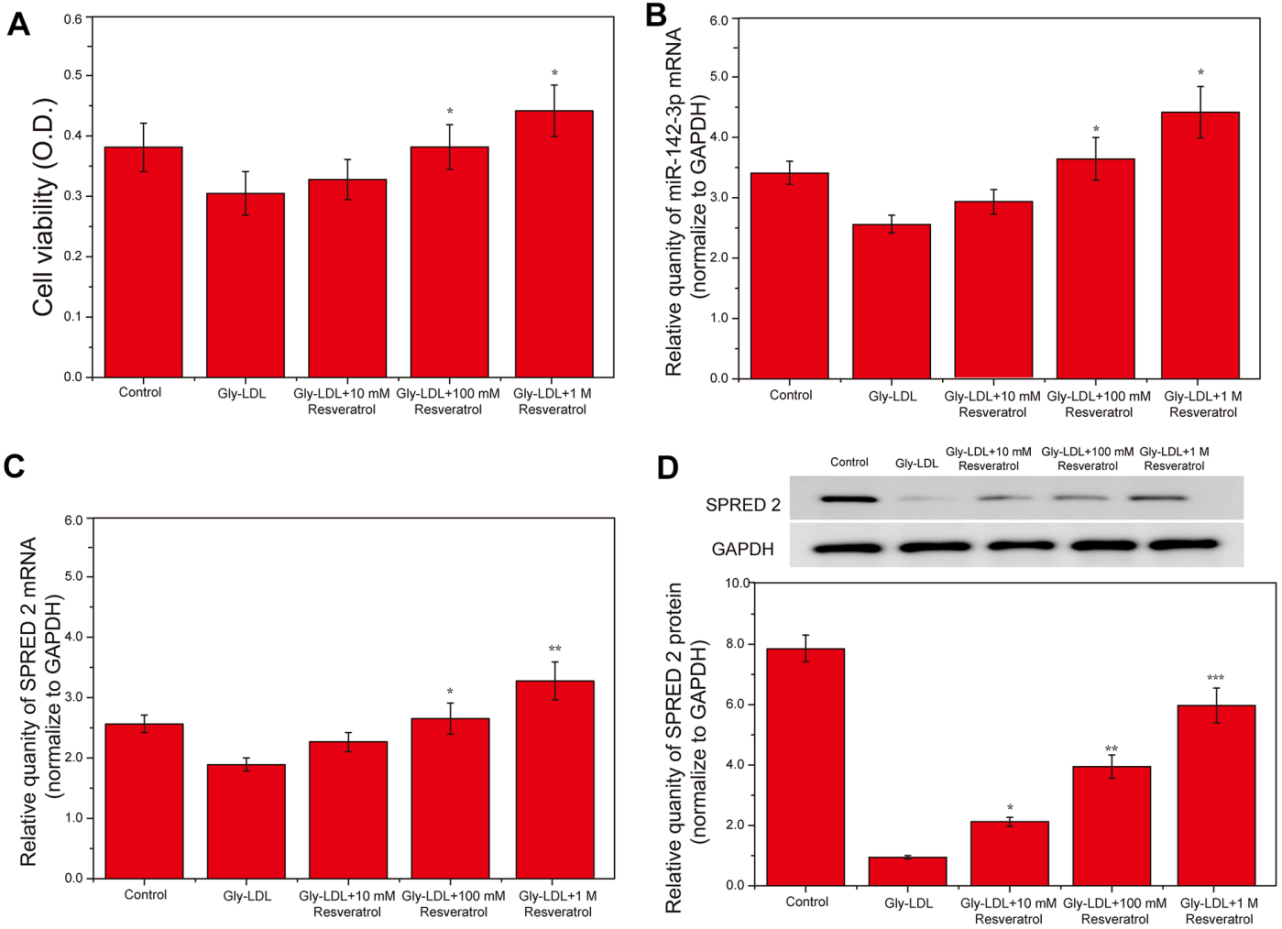
**Resveratrol inhibits the high expression of miR-142-3p in HUVEC induced by Gly-LDL in a dose-dependent manner, and promotes SPRED2 expression and cell proliferation.** (**A**) Cell viability of each group was tested by MTT. (**B**) The expression of miR-142-3p in all groups by PCR. (**C**) The expression of SPRED2 in all groups by western blotting. (**D**) The expression of SPRED2 in all groups by western blotting. *, P < 0.05; **, P < 0.01; ***, P < 0.001.

We successfully established a model of HUVECs induced by Gly-LDL (50 μg/mL). The cells were treated with different doses of resveratrol (10mM, 100mM and 1M). The activity of all groups of cells was tested by MTT, and it was found that Gly-LDL reduced the activity of the cells, while resveratrol showed a dose-dependent increasing in the activity of the cells (Figure 1A, P<0.05). Subsequently, the expression of miR-142-3p in different cells was detected by PCR, and it was found that Gly-LDL caused the lowest expression of miR-142-3p, and different doses of resveratrol increased the expression of miR-142-3p (Figure 1B, P<0.05). At the same time, PCR was used to detect the expression of SPRED2 in different groups, indicating that Gly-LDL reduced the expression of SPRED2; while resveratrol dose-dependently increased the expression of SPRED2 (Figure 1C, P<0.05). Finally, WB detection found that the expression of SPRED2 was lowest in Gly-LDL and resveratrol improved the inhibitory effect of Gly-LDL on SPRED2, showing a dose-dependent (Figure 1D, P<0.05).

### Overexpression of miR-142-3p reverses the effect of resveratrol on Gly-LDL-induced HUVECs proliferation, apoptosis, inflammatory factor secretion and oxidative stress

**Figure 2:**
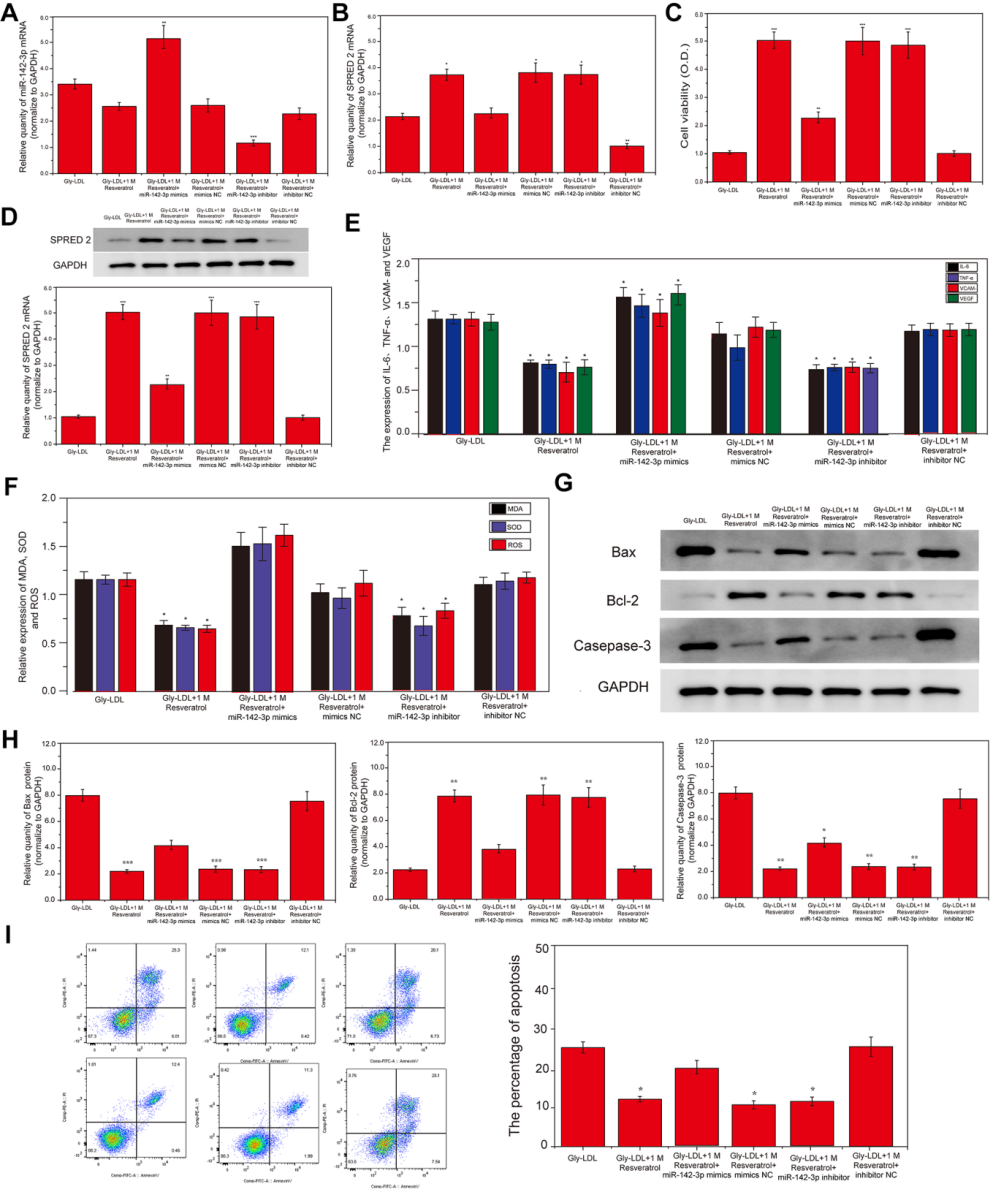
**Overexpression of miR-142-3p reverses the effect of resveratrol on Gly-LDL-induced HUVECs proliferation, apoptosis, inflammatory factor secretion and oxidative stress.** (**A**, **B**) PCR result of overexpression of miR-142-3p and SPRED2. (**C**) Cell viability of each group was tested by MTT. (**D**) The expression of SPRED2 in all groups by western blotting. (**E**) The expression of inflammatory factors IL-6, TNF-α, VCAM- and VEGF. (**F**) The expression of oxidative stress factors MDA, SOD and ROS. (**G**) The expression of caspase-3, BAX and BCL-2 in all groups by western blotting. (**H**) Statistical analysis of Western blotting results. (**I**) The apoptosis of all groups was detected by flow cytometry, and statistical analysis of flow cytometry results. *, P < 0.05; **, P < 0.01; ***, P < 0.001.

1M resveratrol was used to treat cells in all model groups, and miR-142-3p mimics were used to overexpress miR-142-3p, or miR-142-3p inhibitor was used to inhibit the expression of miR-142-3p. The expression of SPRED2 and miR-142-3p in all groups of cells was detected by PCR, and the results showed that the expression of SPRED2 and miR-142-3p was successfully overexpressed or inhibited (Figure 2A, 2B, P<0.05). Cell viability tests found that the 1M resveratrol group, miR-142-3p si-NC and miR-142-3p inhibitor group had significantly higher cell viability than other groups (Figure 2C, P<0.05). Similarly, SPRED2 protein expression was highest in the 1M resveratrol group, miR-142-3p si-NC and miR-142-3p inhibitor group (Figure 2D, P<0.05). Inhibition of miR-142-3p can significantly reduce the expression of oxidative stress factors (MDA, SOD and ROS) and inflammatory factors (IL-6, TNF-α, VCAM- and VEGF) in the cell supernatant (Figure 2E, 2F, P<0.05). The expression of apoptosis-related proteins caspase-3 and BAX was the lowest, while BCL-2 was highest in the 1M resveratrol, miR-142-3p si-NC and miR-142-3p inhibitor groups (Figure 2G, 2H, P<0.05). The apoptosis of all groups was detected by flow cytometry, and it was found that the apoptosis level of 1M resveratrol, miR-142-3p si-NC and miR-142-3p inhibitor groups was significantly lower than that of Gly-LDL + 1M resveratrol + miR-142-3p mimics group (Figure 2I, P<0.05). These results indicated that the overexpression of Mir-242-3P could impair the protective effects of resveratrol on the proliferation, apoptosis, inflammatory factor secretion and oxidative stress of HUVEC cells induced by gly-LDL.

### Overexpression of miR-142-3p reverses the effect of resveratrol on the autophagy and cell cycle of HUVEC cells induced by Gly-LDL

**Figure 3:**
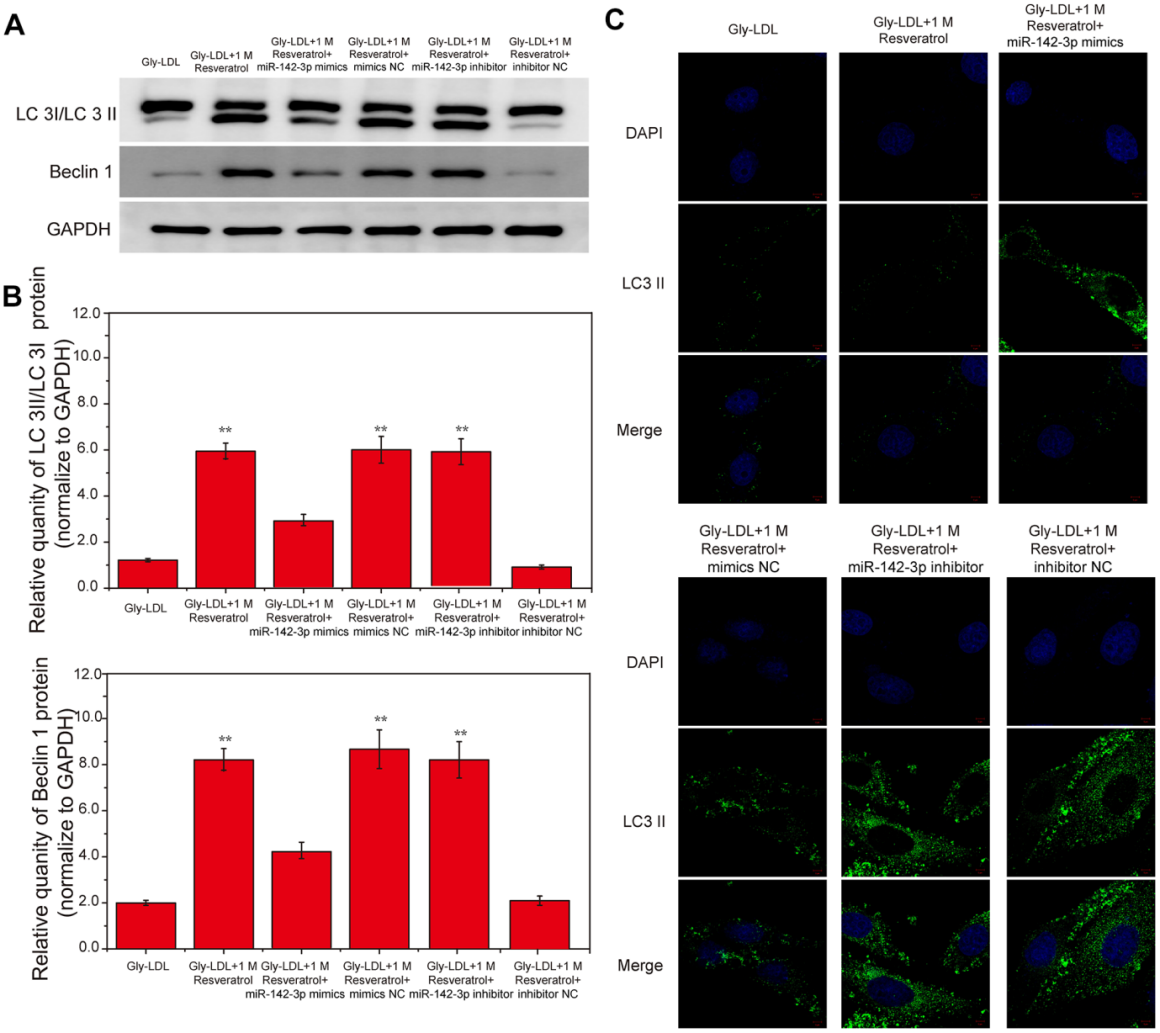
**Overexpression of miR-142-3p reverses the effect of resveratrol on the autophagy and cell cycle of HUVEC cells induced by Gly-LDL.** (**A**) The expression of LC3 II/I and BECLIN1 in all groups by western blotting. (**B**) Statistical analysis of Western blotting results. (**C**) Immunofluorescence detection of LC3. *, P < 0.05; **, P < 0.01; ***, P < 0.001.

Here, we tested the autophagy levels in all groups of cells, the expression of autophagy-related proteins LC3 II/I and BECLIN1 was significantly higher than that of other groups in Gly-LDL + 1M resveratrol, Gly-LDL + 100mM resveratrol + mimics NC and Gly-LDL + miR-142-3p inhibitor groups (Figure 3A, 3B, P<0.05). Immunofluorescence detection verified the results of western blotting, and LC3 fluorescence intensity in the Gly-LDL + miR-142-3p inhibitor group was significantly higher than that in the control group and the Gly-LDL + 1M resveratrol + miR-142-3p mimics group (Figure 3C,P<0.05). The overexpression of miR-142-3p inhibits the autophagy level of model cells to a certain extent.

### Dual luciferase report analysis to detect the binding and regulatory relationship of miR-142-3p and SPRED2

**Figure 4:**
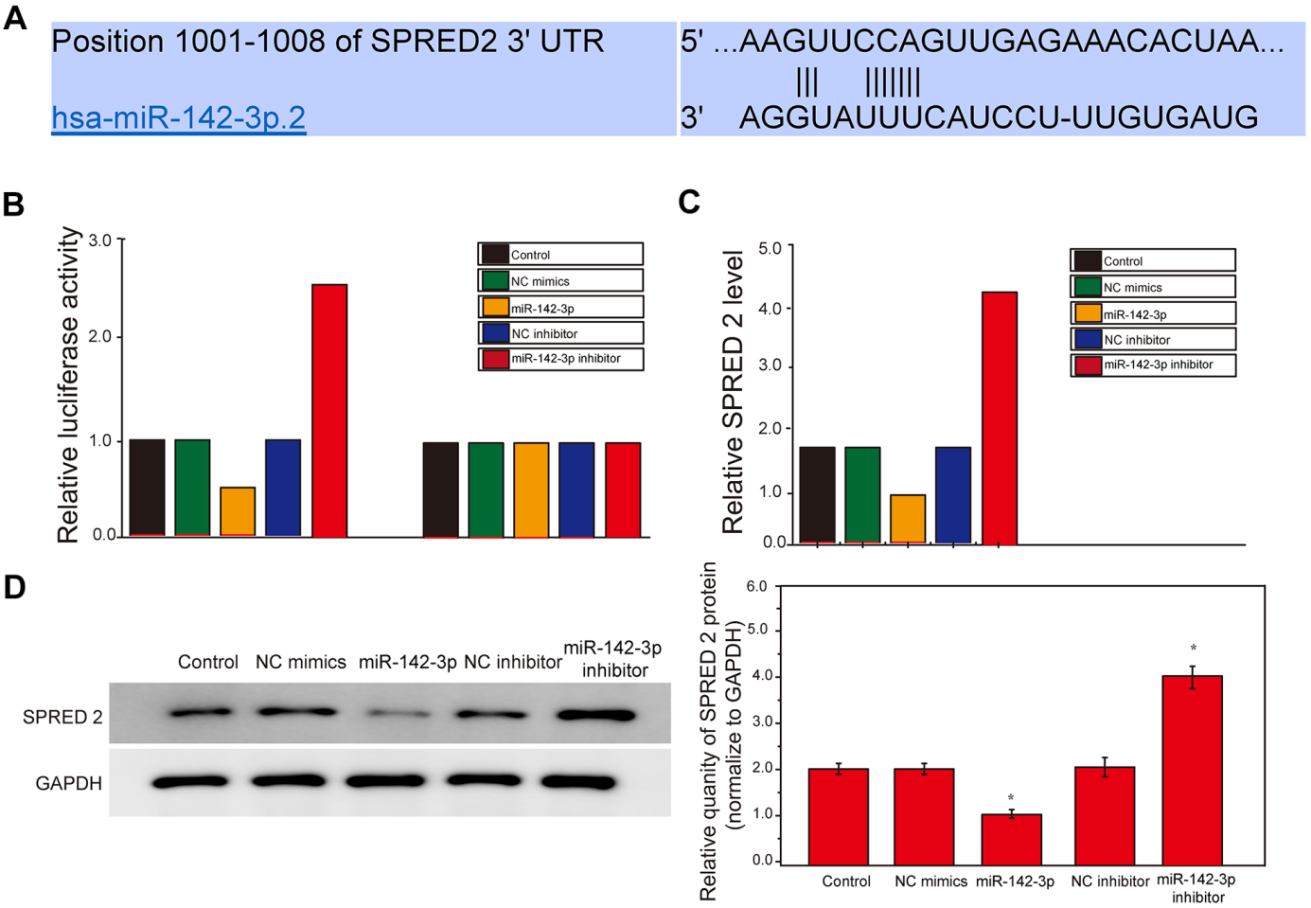
**miR-142-3p and SPRED2 bind to each other and miR-142-3p negatively regulates SPRED2.** (**A**) The position of SPRED2 3’ UTR has-miR--142-3p.2 and Luciferase primer pairs of miR-142-3p. (**B**) Expression of Luciferase Activity in Wild Type and Mutant Type. (**C**) Dual luciferase report analysis detected the relationship between miR-142-3p and SPRED2. (**D**) The expression of Spred2 were detected by western blotting and the statistical analysis of western blotting results. *, P < 0.05; **, P < 0.01; ***, P < 0.001.

In this study, the miR-142-3p gene was overexpressed and inhibited, respectively, and the dual luciferase reporter analysis was used to detect the binding and regulatory relationship between miR-142-3p and SPRED2. SPRED2 3’ UTR has-miR--142-3p.2 is located between 1001-1008 (Figure 4A, P<0.05). The luciferase activity report showed that the wild-type miR-142-3p inhibitor group had the highest luciferase activity and SPRED 2 gene expression (Figure 4C, P<0.05). The results showed that the Gly-LDL + miR-142-3p inhibitor group had a large amount of SPRED2 expression, while the Gly-LDL + 1M resveratrol + miR-142-3p mimics group had SPRED2 expression less than 1, indicating that there is an interaction between miR-142-3p and Spred2 and it is negatively correlated. The results of western blotting also showed that the expression of SPRED2 in the miR-142-3p inhibitor group was the highest, while that in the miR-142-3p mimics group was the lowest (Figure 4D, P<0.05), indicating that Cav1.3 and Spred2 have a strong negative correlation between the proteins.

### Inhibition of SPRED2 reverses the effect of resveratrol on Gly-LDL-induced HUVEC cell proliferation, apoptosis, inflammatory factor secretion and oxidative stress

**Figure 5:**
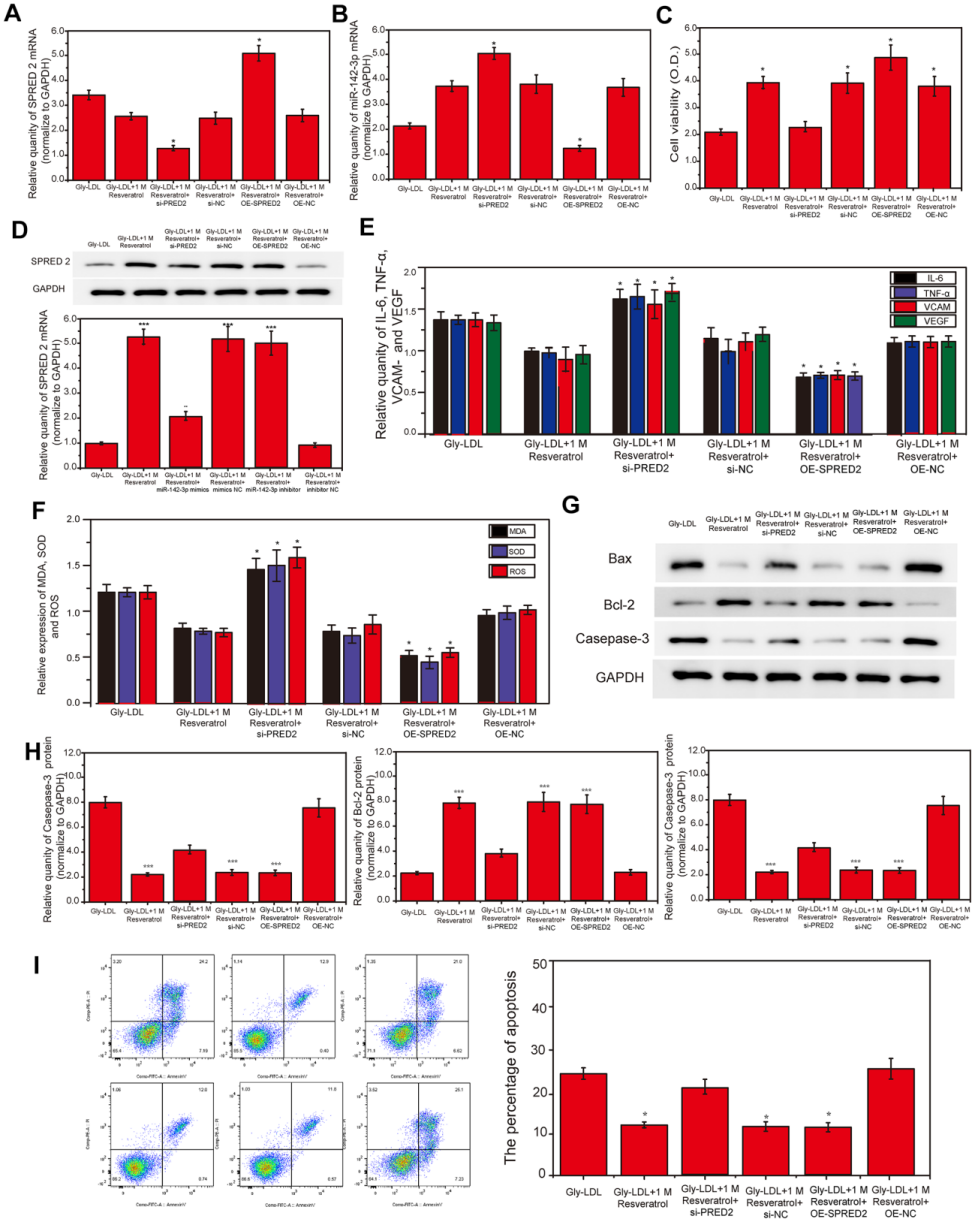
**Inhibition of SPRED2 reverses the effect of resveratrol on Gly-LDL-induced HUVEC cell proliferation, apoptosis, inflammatory factor secretion and oxidative stress.** (**A**, **B**) PCR result of overexpression of miR-142-3p and SPRED2. (**C**) Cell viability of each group was tested by MTT. (**D**) The expression of SPRED2 in all groups by western blotting. (**E**) The expression of inflammatory factors IL-6, TNF-α, VCAM- and VEGF. (**F**) The expression of oxidative stress factors MDA, SOD and ROS. (**G**) The expression of caspase-3, BAX and BCL-2 in all groups by western blotting. (**H**) Statistical analysis of Western blotting results. (**I**) The apoptosis of all groups was detected by flow cytometry, and statistical analysis of flow cytometry results. *, P < 0.05; **, P < 0.01; ***, P < 0.001.

In order to determine the relationship between miR-142-3p and SPRED2, SPRED2 was overexpressed or inhibited in this study. PCR results revealed that miR-142-3p expression in the Gly-LDL OE-SPRED2 group was lower than the other groups, while the expression of miR-142-3p was the highest in the Gly-LDL 1M resveratrol si-PRED2 group. Subsequently, the protein expression results of SPRED2 showed that the Gly-LDL 1M resveratrol and Gly-LDL OE-SPRED2 groups had the highest expression, indicating that the transfection was successful. Similarly, cell viability testing found that overexpression of SPRED2 can promote the proliferation of model cells, while inhibition of SPRED2 reduces the viability of model cells. Inhibition of SPRED2 can significantly increase the expression of oxidative stress factors (MDA, SOD and ROS) and inflammatory factors (IL-6, TNF-α, VCAM- and VEGF) in the cell supernatant (Figure 1E, 1F, P < 0.05). In the 1M resveratrol group, the expressions of apoptosis-related proteins caspase-3, BAX and BCL-2 were the lowest in the Gly-LDL + 100mM resveratrol + si-NC and Gly-LDL + OE-SPRED2 group (Figure 1G, 1H, P <0.05), indicating that the inhibition of SPRED2 can reverse the effects of resveratrol on the proliferation, apoptosis, inflammatory factor secretion and oxidative stress induced by Gly-LDL in HUVEC cells. The apoptosis of all groups was detected by flow cytometry, and it was found that the apoptosis level of Gly-LDL+1M resveratrol, Gly-LDL+SPRED2 si-NC and Gly-LDL+OE-SPRED2 groups was significantly lower than that of Gly-LDL+1M resveratrol+si- SPRED2 group (Figure 5I, P<0.05). These results indicate that the inhibition of SPRED2 reduce the protective effect of resveratrol on the proliferation, apoptosis, inflammatory factor secretion and oxidative stress induced by Gly-LDL in HUVEC cells.

### Inhibition of SPRED2 reverses the effect of resveratrol on the autophagy, senescence and cell cycle of HUVEC cells induced by Gly-LDL

**Figure 6:**
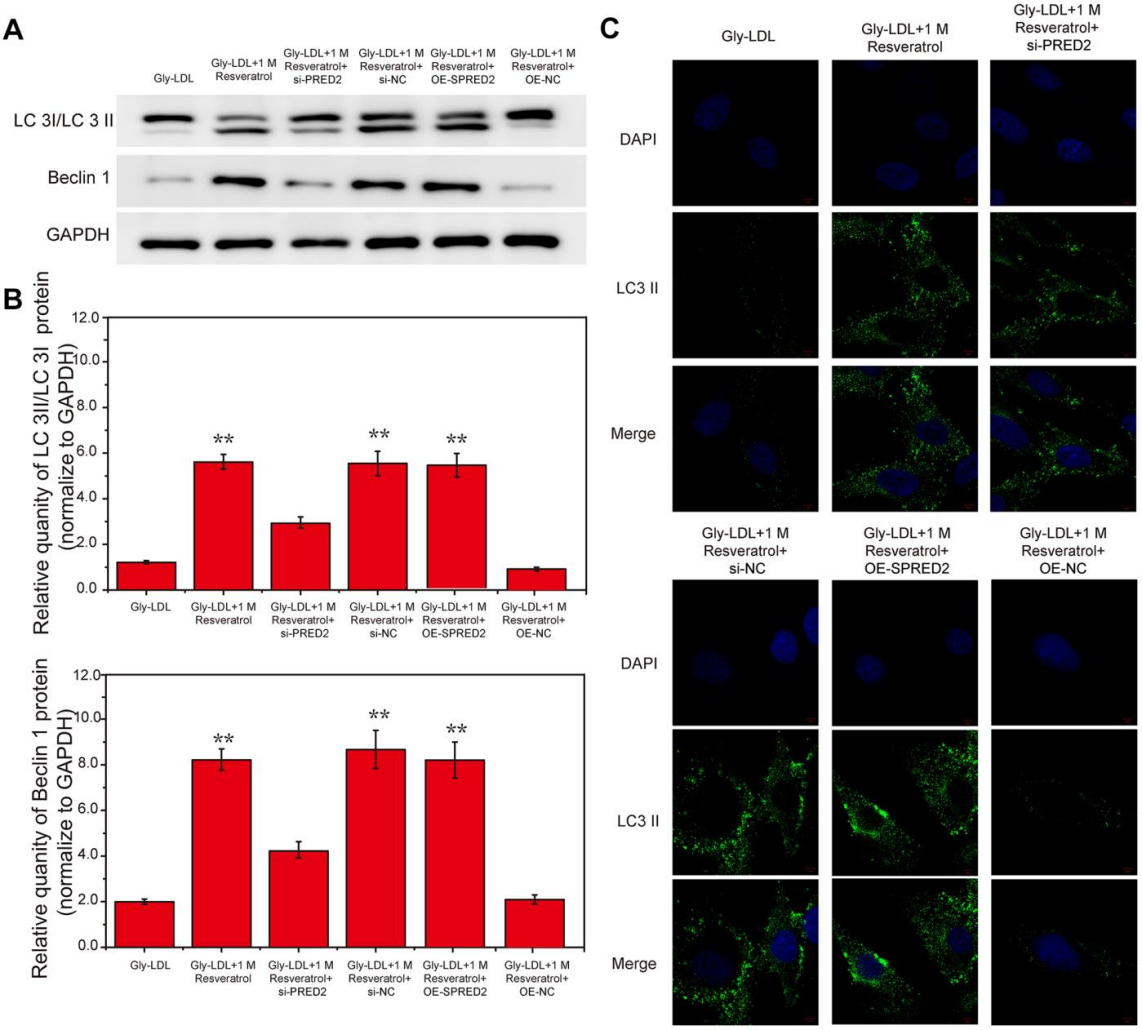
**Inhibition of SPRED2 reverses the effect of resveratrol on the autophagy, senescence and cell cycle of HUVEC cells induced by Gly-LDL.** (**A**) The expression of LC3 II/I and BECLIN1 in all groups by western blotting. (**B**) Statistical analysis of Western blotting results. (**C**) Immunofluorescence detection of LC3. *, P < 0.05; **, P < 0.01; ***, P < 0.001.

Here, we have tested the autophagy level of each group of cells. The expression of autophagy-related proteins LC3 II/I and BECLIN1 in the model group and the Gly-LDL + OE-NC group was higher than the other groups. The LC3 II / I and BECLIN1 expression in the Gly-LDL + 1M resveratrol, Gly-LDL + 100mM resveratrol + si-NC and Gly-LDL + OE-SPRED2 groups was lower than Gly-LDL + 1M Resveratrol + si-PRED2 group (Figure 6A, 6B, P<0.05). The immunofluorescence detection results of LC 3 were consistent with the Western blot results. Inhibition of SPRED2 can increase the level of autophagy and overexpression of SPRED2 can inhibit the level of autophagy in cells.

## DISCUSSION

Hyperglycemia is an important factor involved in the different pathogenesis and pathogenesis of diabetic vascular endothelial function damage. Many studies have shown that hyperglycemia induces oxidative stress and damages vascular endothelium by mediating increased superoxide production. There is also research that high glucose can activate multiple pathways and damage vascular endothelium [13]. Impaired endothelial function will not only cause macrovascular disease and atherosclerosis, but also participate in microvascular disease. Studies have shown that resveratrol can repair the damage of vascular endothelial cells induced by high glucose, but the specific mechanism is unknown [14]. Therefore, this study we have established a Gly-LDL cell model by high glucose, which laid the foundation for cell experiments for the next step.

MiR-142 plays an important role in a variety of tissue injuries. miR-142-5p controls profibrogenic macrophage program under the regulation of L-4 and IL-13. In diabetic nephropathy models, the up-regulation of miR-142-5p inhibits autophagy through up-regulation of BECN1 [15]. Our data has shown that Gly-LDL induces the up-regulation of miR-142-3p, while the addition of different concentrations of resveratrol inhibits the expression of miR-142-3p, indicating that the increase of miR-142-3p will damage blood vessels. Resveratrol protects the function of vascular endothelial cells by inhibiting the expression of miR-142-3p. Although the function of miR-142-3p has been reported in various tissues such as nerves and kidneys [16], its mechanism in vascular endothelial injury is not clear. We have further found that the expression of SPRED2 gradually increases under the action of resveratrol, indicating that SPRED2 may be involved in the repair of vascular endothelial damage.

SPRED2 has recently been found to activate autophagy, and it is also a downstream target of miR-142-3p [17]. Therefore, we overexpressed and suppressed the expression of miR-142-3p in this study. We found that after inhibiting the expression of miR-142-3p, cell viability and SPRED2 expression increased significantly. At the same time, the levels of oxidative stress (MDA, SOD and ROS) and inflammatory factors (IL-6, TNF-α, VCAM- and VEGF) in the cell are reduced, further indicating that low levels of miR-142-3p promotes the repair of vascular endothelial cells. BAX is a water-soluble related protein homologous to BCL-2, it is an apoptosis-promoting gene in the BCL-2 gene family. Overexpression of BAX antagonizes the protective effect of BCL-2 and causes cell death [18]. In this study, the BAX level of the miR-142-3p inhibition group was higher than that of the miR-142-3p overexpression group and the BCL-2 level was lower than that of the overexpression group. Apoptosis detection further illustrates the protective effect of miR-142-3p on vascular endothelial cells. Autophagy plays a protective role in diabetes and high glucose-induced damage [19, 20]. In this study, the level of autophagy in the miR-142-3p inhibitory group increased while the level of autophagy in the miR-142-3p overexpression group decreased. We inferred that miR-142-3p may inhibit SPRED2-mediated autophagy Level, and then induce cell apoptosis, aggravate cell oxidative stress and inflammatory factor secretion, and ultimately promote the development of diabetes.

Interestingly, we found and confirmed the negative regulatory relationship between miR-142-3p and SPRED2 through the luciferase analysis report. Inhibiting the expression of miR-142-3p significantly increased the expression of SPRED2, while the high expression of SPRED2 combined with resveratrol increased the activity of model cells, and reduced intracellular oxidative stress and inflammation levels, and cell apoptosis. This study also showed that the combination of highly expressed resveratrol and SPRED2 promoted the proliferation of vascular endothelial cells, reduced cell apoptosis, and ultimately enhanced the repair ability of blood vessels through Gly-LDL. Finally, we tested the autophagy level of cells after overexpression or suppression of SPRED2. Autophagy is a protective mechanism within the cell that helps the cell to remove foreign objects. Our results were consistent with the previous studies, further indicating that the protective effect of resveratrol combined with high expression SPRED2 on vascular endothelial cells.

This study is the first time to explore the relationship between miR-142-3p and SPRED2 at the cellular level. The effect of resveratrol on the mechanism of high glucose-induced vascular endothelial cells provides a theoretical basis for the occurrence and development of diabetes. guide. This study only explored at the cellular level, and the next step will be to do further research on the mechanism at the animal level.

## CONCLUSIONS

We have systematically studied the effect of the relationship between miR-142-3p and SPRED 2 on vascular endothelial cells induced by Gly-LDL under the action of resveratrol. The increase of miR-142-3p impairs the effect of resveratrol on cell proliferation, apoptosis and autophagy induced by Gly-LDL. SPRED 2 promotes the proliferation and autophagy of resveratrol on vascular endothelial cells damaged by Gly-LDL, and reduces the level of apoptosis, thereby slowing down the damage of vascular endothelial cells under Gly-LDL. In short, miR-142-3p negatively regulates SPRED 2 mediated proliferation, autophagy and apoptosis, and ultimately reduces the ability of resveratrol to repair blood vessels damaged by Gly-LDL.

## MATERIALS AND METHODS

### Culture of HUVECs and establishment of glycated low-density lipoprotein-induced model (Gly-LDL)

This is a basic research. The HUVECs were purchased from Shanghai Institute of Cell Research, Chinese Academy of Sciences, and routinely cultured in 10% fetal bovine serum (FBS)-added Dulbecco's modified eagle medium (DMEM) with low glucose medium containing 1% penicillin-streptomycin. The medium was changed once a day and passage was performed once every 3 days.

After 6 hours of starvation of HUCECs, Gly-LDL was added for intervention (Gly-LDL was dissolved in DMSO with a final concentration of less than 0.1%). Saccharification of LDL: 2mg/ml LDL was added into different concentrations of glucose (group A: 5 mmol/L, group B: 25 mmol/L and group C: 100 mmol/L), and 10^-3^ g/L EDTA and 10^-5^ mol/L BHT (antioxidant) were added, the samples were incubated for 4 weeks to generate Gly-LDL, and dialyzed with Phosphate Buffered Saline (PBS) at 4° C for 48 hours before using.

Different doses of resveratrol (10 mM, 100 mM, 1 M) were treated cells for 24 hours, 48 hours and 72 hours respectively. Cells are grouped into: 1) control group; 2) Gly-LDL model group; 3) model 10mM resveratrol; 4) model 100mM resveratrol; 5) model 1M resveratrol.

Cell transfection: the plasmid of miR-142-3p mimics and miR-142-3p inhibitor were purchased from Shanghai Bioengineering Co., Ltd. Plasmids were transfected into all groups using Lipofectamine3000 (Invitrogen, USA), and the transfection and expression were checked by light microscope and PCR after 24 hours.

### MTT

The cell suspension (100 μL/well) was seeded in a 96-well plate, and the culture plate was placed in an incubator for pre-culture (37° C, 5% CO 2). Different concentrations of drugs were added into the cells, 24 hours later, 10 ul of MTT solution (5 mg/ml) was added to each well, and continue to incubate at 37 degrees for 4-6 hours. The medium was aspirated, 150 μl of DMSO was added to each well, and shaken on a shaker for 10 minutes. All groups were measured by a microplate reader at the absorbance at 570 nm.

### PCR

The cells in all groups were lysed with Trizol, chloroform was used to extract the total RNA, and the kit was used to reverse the RNA to cDNA. SLAN fluorescence quantitative PCR instrument was used to detect the expression of miR-142-3p and SPRED2 in cells. Gene sequence: miR-142-3p: Forward 5′- AGTCAGAACAGAAATACATC-3′, Reverse 5′-TGGCCTCAAGTGATCCTCCCA -3′. SPRED2: Forward 5′- GGA GGCTTTGATGTCGAAGCCCT-3′, Reverse 5′- CCTCCGAAACTACAGCTTCGGGAG-3′. GAPDH: Forward 5′-GTTTACATG TTCCAATATG-3′, Reverse 5′- GTGGGTGTCGCTGTTG AAG-3’.

### Enzyme linked immunosorbent assay (ELISA)

MDA, SOD, ROS, IL-6, TNF-α, VCAM- and VEGF antigens were diluted with coating diluent to an appropriate concentration, 100 μl antigen were added to each well, and incubated at 37° C for 4h. The cells were blocked with 5% BSA at 37° C for 40min and washed with PBS for 3 times/3min. 100μl of the cell supernatant of each group were added and incubated at 37° C for 60min. All groups were washed with PBS and incubated with antibody for 30min. 100μl TMB-Hydrogen Peroxide Urea Solution was added to all wells, kept in the dark at room temperature for 10 minutes, and added the stop solution within 15 minutes to determine the experimental results. The wavelength of 450nm was detected by the microplate reader.

### Western blot analysis

The cells in all groups were lysed with 200 μl of cell lysate and placed on ice for 1 hour. Subsequently, lysing cells were centrifuged at 12,500 rpm for 15 minutes at 4° C. Then transferred the centrifuged supernatant to a clean centrifuge tube. Bicinchoninic acid (BCA) protein quantification kit was used to determine protein concentration and stored the proteins at -80° C. In electrophoresis, the loading concentration was 50 mg per well. After electrophoresis, the membrane was transferred and blocked. SPRED2, caspase-3, BAX, BCL-2, P16, P21, P53, LC3 II/I, beclin1 and GAPDH (1:500, anti-human, Abcam, USA) were diluted and incubated at 4° C overnight. The secondary antibody was incubated in the dark at room temperature for 45 minutes (1:2000, Abcam, USA). Developers were used for development and photography.

### Flow cytometry analysis

1*10^5^ cells were digested with 0.25% trypsin, and centrifuged at 800 rpm for 6 min. 1 ml of PBS was added to resuspend the cells. Propidium Iodide (PI) solution (0.5ml of 50 μg/ml) were added to cells and stained in the dark for 45 min at room temperature. Then using 300 μm nylon mesh to filter the cells into a new centrifuge tube. The filtered cells were inserted into a flow cytometer.

### Dual luciferase reporter assay

The wild-type (WT) SPRED2 containing the predicted binding site with miR-142-3p was purchased and inserted into the RNA expression vector containing pmirGLO dual luciferase to construct the reporter vector pmirGLO-SPRED2 -WT (Promega Corp., Madison, WI, USA). The binding site of miR-142-3p and SPRED2 was mutated using GeneArt site-directed mutagenesis PLUS system (cat. no. A14604; Thermo Fisher Scientific, Inc.). The mutant (MUT) SPRED2 was inserted into the pmirGLO vector to construct the reporter vector pmirGLO-SPRED2-MUT. The constructed reporter vector and miR-142-3p or control mimic were co-transfected into HUVEC, and the cells were cultured for 24-48 hours. The luciferase activity is measured by a dual luciferase reporter gene assay system (Promega, Madison, WI, USA).

### Immunofluorescence

All cells were fixed for 30 min and washed with PBS three times. 5% of bovine serum albumin (BSA) was added to block the cells for 30 min, and washed three times with PBS. Primary antibody (LC3 II, 1:400, ab192890, Abcam, USA) were added to the cells and incubated at 4° C for 12 h. The cells were washed with PBS three times. Secondary antibody (1:2000, ab150113, Abcam, USA) were added and incubated at room temperature for 30 minutes at dark. The cells were observed by an optical microscope.

### Statistical analysis

All data were analyzed using SPSS 17.0 software, and the data was expressed as mean ± standard deviation (SD). Prism 5.0 software was used for One-way ANOVA or Student t test for differences between within groups and groups. And all experiments were carried out independently three times. P<0.05 was considered to have a significant statistical difference.

### Ethics approval and consent to participate

The research protocol was reviewed and approved by the Ethical Committee and Institutional Review Board of the Minhang Hospital, Fudan University.

### Consent to publish

All of the authors have Consented to publish this research.

### Availability of data and materials

The data are free access to available upon request.
